# SDC4-rs1981429 and ATM-rs228590 may provide early biomarkers of breast cancer risk

**DOI:** 10.1007/s00432-022-04236-2

**Published:** 2022-09-24

**Authors:** Sofia I. Vuorinen, Rachel K. Okolicsanyi, Martina Gyimesi, Jacob Meyjes-Brown, Deepa Saini, Son H. Pham, Lyn R. Griffiths, Larisa M. Haupt

**Affiliations:** Stem Cell and Neurogenesis Group, Genomics Research Centre, Centre for Genomics and Personalised Health, School of Biomedical Sciences, Queensland University of Technology (QUT), 60 Musk Ave, Kelvin Grove, QLD 4059 Australia

**Keywords:** Heparan sulfate proteoglycans, Breast cancer, Single-nucleotide polymorphism, Phosphatidyl-inositol-3-kinase/Protein kinase B pathway, Syndecan-4, ATM serine/threonine kinase

## Abstract

In Australia, 13% of women are diagnosed with breast cancer (BC) in their lifetime with approximately 20,000 women diagnosed with the disease in 2021. BC is characterised by complex histological and genomic influences with recent advances in cancer biology improving early diagnosis and personalised treatment interventions. The Phosphatidyl-inositol-3-kinase/Protein kinase B (PI3K/AKT) pathway is essential in apoptosis resistance, cell survival, activation of cellular responses to DNA damage and DNA repair. Heparan sulfate proteoglycans (HSPGs) are ubiquitous molecules found on the cell surface and in the extracellular matrix with essential functions in regulating cell survival, growth, adhesion and as mediators of cell differentiation and migration. HSPGs, particularly the syndecans (SDCs), have been linked to cancers, making them an exciting target for anticancer treatments. In the PI3K/AKT pathway, syndecan-4 (*SDC4*) has been shown to downregulate AKT Serine/Threonine Kinase (*AKT1*) gene expression, while the ATM Serine/Threonine Kinase (*ATM*) gene has been found to inhibit this pathway upstream of *AKT*. We investigated single-nucleotide polymorphisms (SNPs) in HSPG and related genes *SDC4*, *AKT1* and *ATM* and their influence on the prevalence of BC. SNPs were genotyped in the Australian Caucasian Genomics Research Centre Breast Cancer (GRC-BC) population and in the Griffith University–Cancer Council Queensland Breast Cancer Biobank (GU-CCQ BB) population. We identified that *SDC4-*rs1981429 and *ATM-*rs228590 may influence the development and progression of BC, having the potential to become biomarkers in early BC diagnosis and personalised treatment.

## Introduction

Breast cancer (BC) is the second leading cause of death in the female population, affecting approximately 1 in 7 women and 1 in 700 men worldwide (Australian Institute of Health and Welfare 2020; Sung et al. 2021). BC is characterised by complex histological and genomic influences throughout both early and late stages of disease development, with 20% of cases estimated to be hereditary (Bertucci et al. 2019; McCart Reed et al. 2021). Treatment is often aggressive and debilitating, involving mastectomies, chemotherapy and radiotherapy (Veronesi et al. 2006). Therefore, early detection, diagnosis and intervention strategies are integral to reducing mortality and morbidity rates, with pre-metastatic BC having a 5-year survival rate of approximately 90% (Kamangar et al. 2006; Network 2012). BC originates from mammary epithelial cells, developing from abnormal cell growth and proliferation (Zhang et al. 2017). Subtypes of BC include luminal A, luminal B, basal-like and human epidermal growth factor receptor 2 (HER2) positive and triple-negative breast cancer (TNBC) (Arnone et al. 2010; Bareche et al. 2018).

**Fig. 1:**
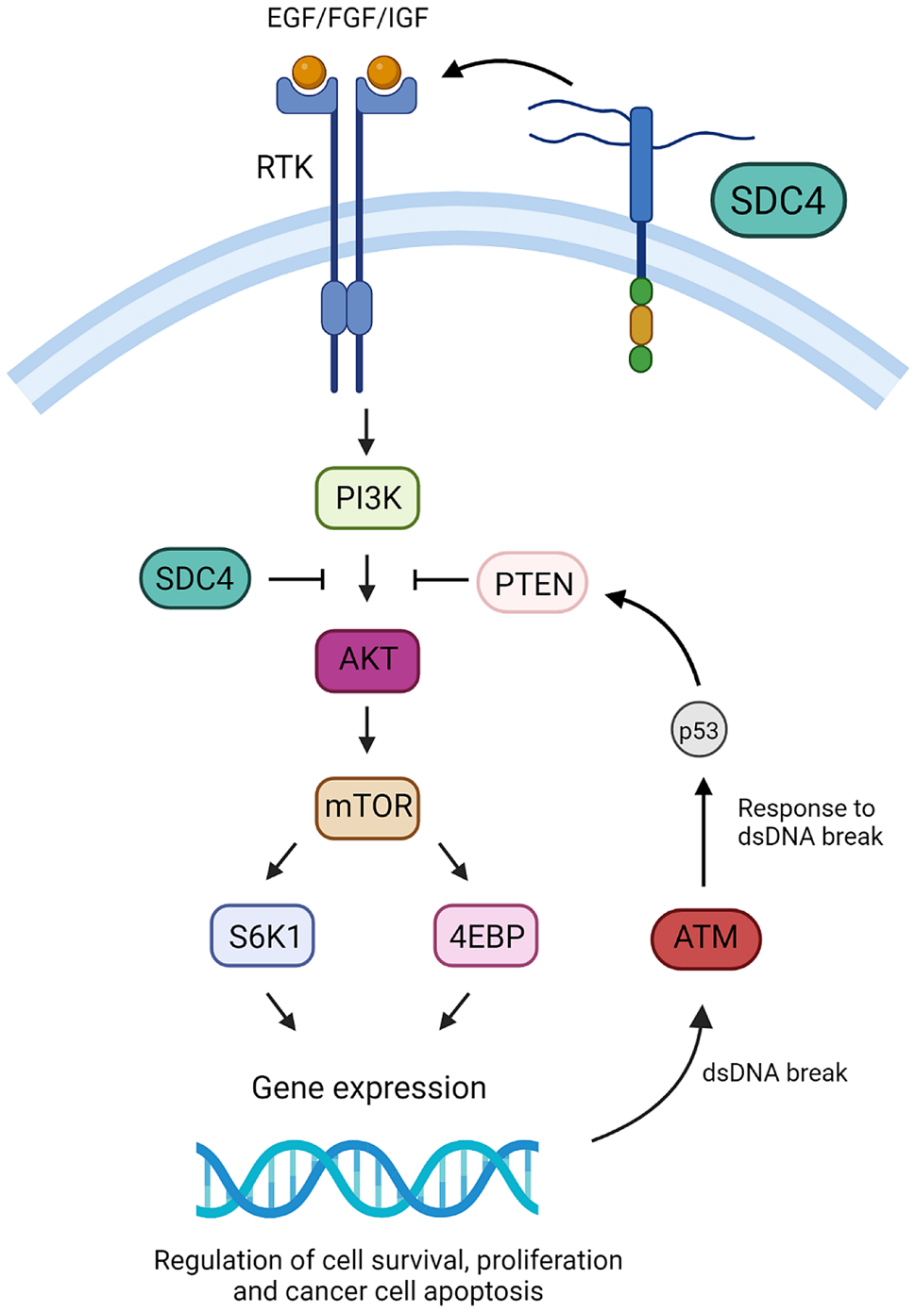
Proposed model of the Phosphoinositide 3-kinase/AKT Serine/Threonine-Protein Kinase (PI3K/AKT) signalling pathway: Phosphoinositide 3-kinases (PI3K) are activated by tyrosine kinase receptors (RTK), which are stimulated by several growth factors, including Epidermal Growth Factor (EGF), Fibroblast Growth Factor (FGF) and Insulin-like Growth Factor (IGF). Syndecan-4 (SDC4) forms connections between the extracellular matrix (ECM) and signalling proteins, modulating growth factor binding to RTK. PI3K binds to its receptor, triggering a chain of events that recruits AKT Serine/Threonine-Protein Kinase (AKT) to the cell membrane. AKT targets molecules such as mammalian target of rapamycin (mTOR). mTOR phosphorylates downstream proteins, including Ribosomal protein S6 kinase beta-1 (S6K1) and 4E-binding protein (4EBP). All these steps culminate in gene expression regulation, and ultimately cellular proliferation, apoptosis, survival and angiogenesis, as well as other physiological functions. The Phosphatase and Tensin homolog (PTEN) enzyme and SDC4 proteins inhibit the pathway, normalising cellular proliferation and metabolism. Double-stranded DNA (dsDNA) breaks lead to the activation of ATM serine/threonine kinase (ATM), which stimulates the tumour protein p53 (p53). p53 triggers PTEN, which in turn inhibits the downstream regulation of AKT and mTOR, reducing gene expression. Aberrations in the PI3K/AKT pathway have been linked to breast cancer

Single-nucleotide polymorphisms (SNPs) may affect the functions of key signalling pathways, increasing the risk of BC development (Gao et al. 2019). The Phosphatidyl-inositol-3-kinase/Protein kinase B (PI3K/AKT) signalling pathway contributes to cell survival, proliferation, angiogenesis and metabolism, as shown in Fig. 1 (Araki and Miyoshi 2018; Wu et al. 2020). It is essential to cell survival, apoptosis resistance, activation of cellular responses to DNA damage and repair of drug-induced DNA damage (Araki and Miyoshi 2018; Xu et al. 2020). Syndecan-4 (*SDC4*) inhibits *AKT* expression in the pathway and mediates activation of several co-factors such as protein kinase C (PKC), which regulates subsequent pathway signalling (Bertucci et al. 2019; Ju and Simons 2013; Mochizuki et al. 2020). The recognised oncogene AKT Serine/Threonine Kinase 1 (*AKT1*) regulates PI3K/AKT pathway functions via serine and/or threonine phosphorylation (Bertucci et al. 2019; Li et al. 2018). *AKT1* mutations disrupt the pathway, increasing the risk of BC (Bonin et al. 2019; Hinz & Jucker 2019). Phosphatidyl-inositol-3-kinase (PI3K) is directly activated by cell surface receptors such as receptor tyrosine kinase (RTK), stimulated by growth factors including Epidermal Growth Factor (EGF), Fibroblast Growth Factor (FGF), Vascular Endothelial Growth Factor (VEGF) and Insulin-like Growth Factor (IGF), mediating cell growth, survival, differentiation, glucose transport and metabolism (Corti et al. 2013; Gross and Rotwein 2016; Karczewski et al. 2020; Machiela and Chanock 2015; Schadt et al. 2008). Activation of PI3K recruits AKT to the cell membrane (Corti et al. 2013; Gross and Rotwein 2016; Karczewski et al. 2020; Machiela and Chanock 2015; Schadt et al. 2008). SDC4 forms connections between the extracellular matrix (ECM) and various signalling proteins, such as the AKT1 and FGF1 proteins and modulates growth factor binding to RTK (Elfenbein and Simons 2013; Mochizuki et al. 2020). SDC4 may also have an inhibitory role regulating AKT phosphorylation (Ju and Simons 2013). AKTs main target molecule is the mechanistic target of rapamycin (mammalian target of rapamycin; mTOR), which regulates cellular growth and nutrition by phosphorylating the Ribosomal protein S6 kinase beta-1 (S6K1) and 4E-binding (4EBP) proteins (Gotting et al. 2020; Xu et al. 2020). The ATM protein, encoded by the ATM serine/threonine kinase (ataxia–telangiectasia mutated; *ATM*) gene, regulates glucose homeostasis and is essential for the phosphorylation of AKT. Double-stranded DNA (dsDNA) breaks activate ATM and with it the pro-apoptotic tumour protein p53 (p53), as shown recently (Hwang et al. 2020). p53 consequently activates Phosphatase and Tensin homolog (PTEN), which inhibits the pathway, downregulating cellular proliferation and metabolism (Bonin et al. 2019; Chen et al. 2005; Marty et al. 2008). Each of these steps culminate in differential gene expression and the regulation of several essential physiological functions (Davis et al. 2014; Xu et al. 2020). Dysregulation of this complex pathway has also been linked to chemotherapy resistance, such as the monoclonal antibody Trastuzumab (Herceptin) in HER2-overexpressing BC (Liu et al. 2020; Paplomata and O'Regan 2014; Yu et al. 2015). Recent studies show that inhibition of the PI3K/AKT pathway shows strong potential as a treatment avenue for BC, specifically, HER2 positive subtypes (Kaklamani et al. 2019; Verret et al. 2019). This study aimed to investigate whether the presence of minor alleles in the SDC4, AKT1 and ATM genes alter the function of the PI3K/AKT pathway, affecting the risk of BC development.

Heparan sulfate proteoglycans (HSPGs) are heavily glycosylated macromolecules found in the ECM, on the cell surface and in the cytoplasm (Okolicsanyi et al. 2014). HSPGs are composed of a core protein with several glycosaminoglycan (GAG) side chains covalently bound to the core protein. These proteins undergo a complex temporal biosynthesis process within the Golgi apparatus determining the nature of the GAG chains, specifying them as heparan sulfate (HS), dermatan sulfate (DS), chondroitin sulfate (CS) or keratan sulfate (KS) (Christianson and Belting 2014; Couchman et al. 2015). Syndecans (SDCs) have HS side chains and may also carry CS side chains. These side chains bind to a wide variety of protein ligands, such as cytokines, enzymes, growth factors and protein structures within the ECM (Huang et al. 2020; Sugahara and Kitagawa 2002) HSPGs regulate cell survival, growth and adhesion and mediate cell differentiation and migration by binding to several cell adhesion and matrix molecules, proteases and growth factors (Bishop et al. 2007; Knelson et al. 2014). HSPGs, including the SDC1 and SDC4 proteins, have been linked to metabolism, infectious diseases and cancers including BC (Christianson and Belting 2014; Huang et al. 2020; Sugahara and Kitagawa 2002). Imbalances in *SDC4* expression have also been linked to melanomas, liver carcinomas and mesotheliomas (Couchman et al. 2015). *SDC4* overexpression was found to contribute to BC invasiveness, although a clear mechanism of the effects observed in BC has not yet been identified (Couchman et al. 2015; Okolicsanyi et al. 2015). Due to this, the SDCs present an exciting target for macromolecular drug therapies. Understanding the interactions between HSPG-related genes in pathways, such as the PI3K/AKT pathway, and cancer development facilitates the identification of key signalling events to aid early diagnosis and targeted BC treatments.

The PI3K/AKT pathway provides an interesting avenue for BC studies, as controlling and inhibiting aberrant activation may provide a potential target for anticancer treatments. SNPs are known to be important markers in disease development and progression. Here we examined two *SDC4*, two *AKT1* and two *ATM* SNPs for their roles in BC susceptibility in an Australian Caucasian population. These SNPs have not been studied in BC previously; however, various *SDC4* SNPs have previously been linked to melanomas, bladder carcinomas, osteosarcomas, colon, testicular and haematological malignancies, as well as cervical and ovarian cancers (Barbouri et al. 2014; Okolicsanyi et al. 2015), while *ATM* gene SNPs have been linked to breast, ovarian and pancreatic cancers (Gao et al. 2019; Renault et al. 2018). While genetic tests often require an invasive blood test, detecting SNPs via genotyping can be conducted by simply collecting a saliva sample. Simplifying risk screening and diagnostic testing will improve the chances for early intervention and compliance. In addition, improving diagnostics is likely to enhance drug trialling approaches. This study aimed to examine SNPs in the gene encoding the HSPG core protein, *SDC4*, and four SNPs in the HSPG-related genes *AKT1* and *ATM* in the PI3K/AKT pathway for associations with BC risk.

## Methods

### Population demographics

#### Genomics Research Centre (GRC) breast cancer (GRC-BC) case–control cohort

The Genomics Research Centre (GRC) breast cancer (GRC-BC) population consisted of 278 BC case samples and 207 age and sex-matched controls of Caucasian (Northern European) origin. All study participants were Queensland residents diagnosed with histologically confirmed invasive BC. Blood collection for DNA isolation and clinical and demographic information were gathered from the Queensland Cancer Registry and diagnosis and treatment data were obtained via medical records and telephone interviews with participants. The control population included participants with no personal or familial history of BC between the ages of 32 and 88 years. The case population has an average age of 57.5 years at collection, while the control samples have an average age of 53.4 years at time of collection. Certain types of BC, including TNBC, are more prevalent in patients aged 35–45 years (Szollár et al. 2019) and 45 years has been established as an appropriate cut off age for BC studies (Nie et al. 2017). Due to the distribution of ages including the majority of cases and controls in the GRC-BC population being over the age of 45 years, the cut off age of 45 years was selected to ensure sufficient power in sample number for analysis. A summary of the numbers of males and females and their ages can be found in Table 1. There is no BC subtype information for the patients in the GRC-BC case–control cohort, therefore, analysis by molecular subtype could not be conducted. Participants were recruited through the GRC between 1997 and 2018.

**Table 1:** Genomics Research Centre (GRC) breast cancer (GRC-BC) case–control cohort population demographics

	Age			Sex		
	≤ 45	> 45	Age not recorded	Male	Female	Sex not recorded
Cases (*n* = 278)	18	151	109	3	234	41
Controls (*n* = 207)	17	111	79	3	175	23

### Griffith University–Cancer Council Queensland Breast Cancer Biobank (GU-CCQ BB) Cohort

The Griffith University–Cancer Council Queensland Breast Cancer Biobank (GU-CCQ BB) population was used as a replication population and consisted of 372 case samples (Youl et al. 2011). The samples were collected by the GRC in collaboration with Cancer Council Queensland as part of a 5-year population-based longitudinal study of women with BC. Study participants were residents of Queensland with a histologically confirmed diagnosis of invasive BC. Sample collection commenced in 2011 with women aged 33–80 years with an average age 60.2 years. Clinical and demographic information was acquired from the Queensland Cancer Registry and medical record collection and telephone interviews were conducted to acquire diagnostic and treatment information. A summary of the numbers of males and females and their ages can be found in Table 2. The same analysis age parameters and cutoffs were used for the GU-CCQ BB population (Table 1).

**Table 2:** Griffith University–Cancer Council Queensland Breast Cancer Case Only Biobank demographics

	Age			Sex		
	≤ 45	> 45	Age not recorded	Male	Female	Sex not recorded
Cases (*n* = 377)	31	346	0	0	352	25

Control data for the GU-CCQ BB population were extracted from the 1000 Genomes Project for *SDC4*-rs1981429, *SDC4*-rs2251252, *AKT1*-rs10138227, *AKT1*-rs2498794 and *ATM*-rs228590 (Genomes Project Consortium et al. 2015). The data extracted from the 1000 Genomes Project consisted of 404 samples of non-Finnish European origin, including both females and males to accurately match the GU-CCQ BB demographic data.

While control data were not available for *ATM*-rs35098825 in the 1000 Genomes Project due to the rarity of the SNP, the Genome Aggregation Database (gnomAD) database (Karczewski et al. 2020) contained allelic data for this SNP; therefore, gnomAD was used to provide control data for the analysis of *ATM*-rs35098825. gnomAD was also used for a larger analysis for *SDC4*-rs1981429, *SDC4*-rs2251252, *AKT1*-rs2498794, *AKT1*-rs10138227and *ATM*-rs228590 to corroborate the genotypic analysis generated through the 1000 Genomes Project. The gnomAD database did not provide similar demographic information to the 1000 Genomes Project; hence, both databases were utilised for independent GU-CCQ BB case data analyses. The analysis through gnomAD replicated the findings found in the 1000 Genomes Project data.

### DNA extraction and quantification

DNA was extracted from blood by salting-out methods as described by Chacon-Cortes et al. (Chacon-Cortes et al. 2012). Sample DNA quality and quantity was assessed and measured using the NanoDrop™ 8000 Spectrophotometer (Thermo Fisher Scientific, Australia). Samples with a 260/280 ratio of approximately 1.8 were diluted to 10 ng/μL using sterile nuclease-free water, calculated depending on the initial sample concentration. Samples that had degraded or evaporated with a concentration of less than 10 ng/μL were excluded from the study to comply with sample quality requirements. 40 ng of template DNA was used for each polymerase chain reaction (PCR) reaction for restriction fragment length polymorphism (RFLP) and 30 ng was used for high-resolution melt (HRM) reactions.

### Primers and enzyme selection

Primers were designed for RFLP and HRM using NCBI Primer-BLAST (Ye et al. 2016). RFLP was utilised to genotype *SDC4*-rs2251252 in the GRC-BC case–control cohort population, while HRM was utilised to genotype *SDC4*-rs2251252 in the GU-CCQ BB population. HRM was used to genotype *SDC4*-rs1981429, *AKT1*-rs2498794, *AKT1*-rs10138227, *ATM*-rs228590 and *ATM*-rs35098825 in both populations. Primers sequences and annealing temperatures (*T*_a_) for each primer can be found in Table 3. All primers were designed with an optimal GC content between 40 and 60% and an optimal *T*_a_ around 60 °C.

**Table 3:** Assay details and single-nucleotide polymorphism (SNP) information, including amplicon length in base pairs (bp), annealing temperature (*T*_*a*_) and minor and major alleles as well as genotyping method as either restriction fragment length polymorphism (RFLP) or high-resolution melt (HRM). The accession numbers show that all SNPs investigated are located in non-coding regions

Gene	SNP number	Forward primers	Reverse primers	Chr	Chr position	Amplicon length (bp)	Variation	*T*_*a*_ (°C)	Accession number	Assay type
*SDC4*	rs1981429	GGGCCAGACATTGCTCTAAT	TGTGACCCTGGGCAAGTTAC	20	45,347,053	131	A > C	57	NC_000020.10	HRM
*SDC4*	rs2251252	GAAGCAGGAGGCAAGAACAA	GCTGCAGTACTCCCCAAAAG	20	45,342,717	117	G > A	57	NC_000020.11	HRM
*SDC4*	rs2251252	GCCAGAGAAAACTGGAGCAG	ACCTGTGGCACTCAAAGGTC	20	45,342,717	428	G > A	60	NC_000020.11	RFLP
*AKT1*	rs2498794	CATCTGTATGTGGCAGGGCT	AGAGCTACTTGGAGGGGAGG	14	104,778,914	146	A > G	57	NC_000014.9	HRM
*AKT1*	rs10138227	GGAAGACAGGACCAGGATGC	CAGGAGGTTTTTGGGCTTGC	14	104,793,369	78	C > T	57	NC_000014.9	HRM
*ATM*	rs228590	AGATGGCTCTGATTCTCTTCTCCT	GCGGAAGTTGTAATAGTGTTGGG	11	108,225,414	85	C > T	57	NC_000011.10	HRM
*ATM*	rs35098825	AGGAGCTTCCTGGAGAAGAGT	AGCTTAACAGAACACATCAGTTATT	11	108,271,114	136	G > A	57	NC_000011.10	HRM

RFLP was done by digesting the PCR product into two separate fragments. The University of California, Santa Cruz (UCSC) Genome Browser was used for in silico PCR, and NEBcutter2 was used to identify the *Bts*CI restriction enzyme (2,000 SI units; 20,000 units/mL) (New England Biolabs, Catalog#R0647S) recognising the SNP sequence GGATG which was then used to digest the *SDC4*-rs2251252 fragment, cleaving the 428 bp PCR product into 255 bp and 173 bp fragments in the presence of the C allele only (Vincze et al. 2003).

### Polymerase chain reaction (PCR)

DNA fragments were amplified using the following reaction conditions: 40 ng of DNA was amplified with 1X PCR buffer, 200 nM each forward and reverse primers (IDT, USA), 200 μM dNTPs (NEB, Australia), 1.75 mM MgCl_2_, and 0.017 U GoTaq Flexi DNA Polymerase (Promega, Australia) in a 15 μL reaction. The PCR reaction was repeated twice to obtain sufficient amplification for RFLP enzyme digestion.

Cycling conditions consisted of a denaturation step at 95 °C for 2 min, followed by 30 cycles of 95 °C for 1 min, T_a_ for 1 min and extension at 72 °C for 1 min. The final extension step was held for 2 min at 72 °C. PCR products were analysed on a 2% agarose gel in 1X Tris–acetate–EDTA (TAE) with ethidium bromide (EtBr) at 90 V for 30 min to confirm amplification of the correct size fragment for each amplicon. A 100 bp DNA ladder (NEB Australia) was run alongside the samples to indicate sizing. PCR products were imaged under UV light to determine adequate amplification.

### Restriction fragment length polymorphism analysis (RFLP)

Genotyping was performed using RFLP analysis. Following PCR cycling, approximately 1 μg (6 μL) PCR product was digested with 7 U *Bts*CI enzyme for 1 h at 50 °C with 1X reaction buffer in a 15 μL reaction and then cooled down to 4 °C until collection for gel electrophoresis.

### Agarose gel electrophoresis

Following PCR amplification, fragments were analysed using 2% agarose gels with added EtBr in 1X TAE buffer at 90 V for 30 min to confirm correct amplicon size. For genotyping analysis using RFLP, the digested PCR fragments were analysed using a 4% agarose gel with added EtBr in 1X TAE at 70 V for 1 h 45 min for improved resolution. A 100 bp DNA ladder was included to approximate sizing of digested DNA fragments, imaged under UV light.

### High-resolution melt (HRM)

HRM analyses were performed using the Rotor-Gene® Q (Qiagen, Australia) and Applied Biosystems QuantStudio 7 Flex Real-Time PCR System (Thermo Fisher Scientific, Australia). All DNA case–control samples were amplified in duplicate. A positive control representing each genotype was identified for *SDC4*-rs1981429, *SDC4*-rs2251252, *AKT1*-rs2498794, *AKT1*-rs10138227 and *ATM*-rs228590 from samples not included in the studied populations. For *ATM*-rs35098825, only a homozygous (GG) and rare heterozygous (AG) positive control were utilised; a minor allelic homozygote (AA) was not found.

HRM reaction conditions amplified 30 ng of DNA with 1X reaction buffer, 1.5 mM MgCl_2_, 200 nM each of forward and reverse primers, 200 nM dNTPs, 48 μM Syto®9 and 0.05 U GoTaq HotStart DNA polymerase in a 15 μL reaction. The Rotor-Gene® Q system cycling conditions were as follows: denaturation and HotStart polymerase activation at 95 °C for 10 min followed by 40 cycles of 94 °C for 15 s, T_a_ for 1 min and a final melt between 72 °C and 85 °C, rising by 0.1 °C each step with a 2 s wait afterwards.

### Sequence validation using Sanger sequencing

Sequence validation was performed using the BigDye® Terminator (BDT) v3.1 Cycle sequencing kit (Thermo Fisher Scientific, Australia). The concentration of PCR product was estimated to be approximately 40 ng/μL using agarose gel electrophoresis and adjusted for optimal sequencing conditions. Then, the PCR product was cleaned with ExoSAP-IT® (Affymetrix) following manufacturer’s instructions. Separate forward and reverse reactions were prepared for each sample using the BDT sequencing kit. Samples were then cleaned using a standard ethanol precipitation method (Tajouri et al. 2005), followed by drying and resuspension in water prior to sequence detection on the POP-7™ Polymer for 3500/3500xL Genetic Analyzer.

### Statistical tests

Deviations between observed and expected frequencies were calculated using the Hardy–Weinberg equilibrium (HWE) (Kalmes and Huret 2001) with Chi-squared analysis used to test for statistically significant differences between the case and control populations. The odds ratio (OR) was used to calculate indicated disease risk at a confidence interval of 95%.

### LD analysis of SDC4 and AKT1 SNPs

**Fig. 2:**
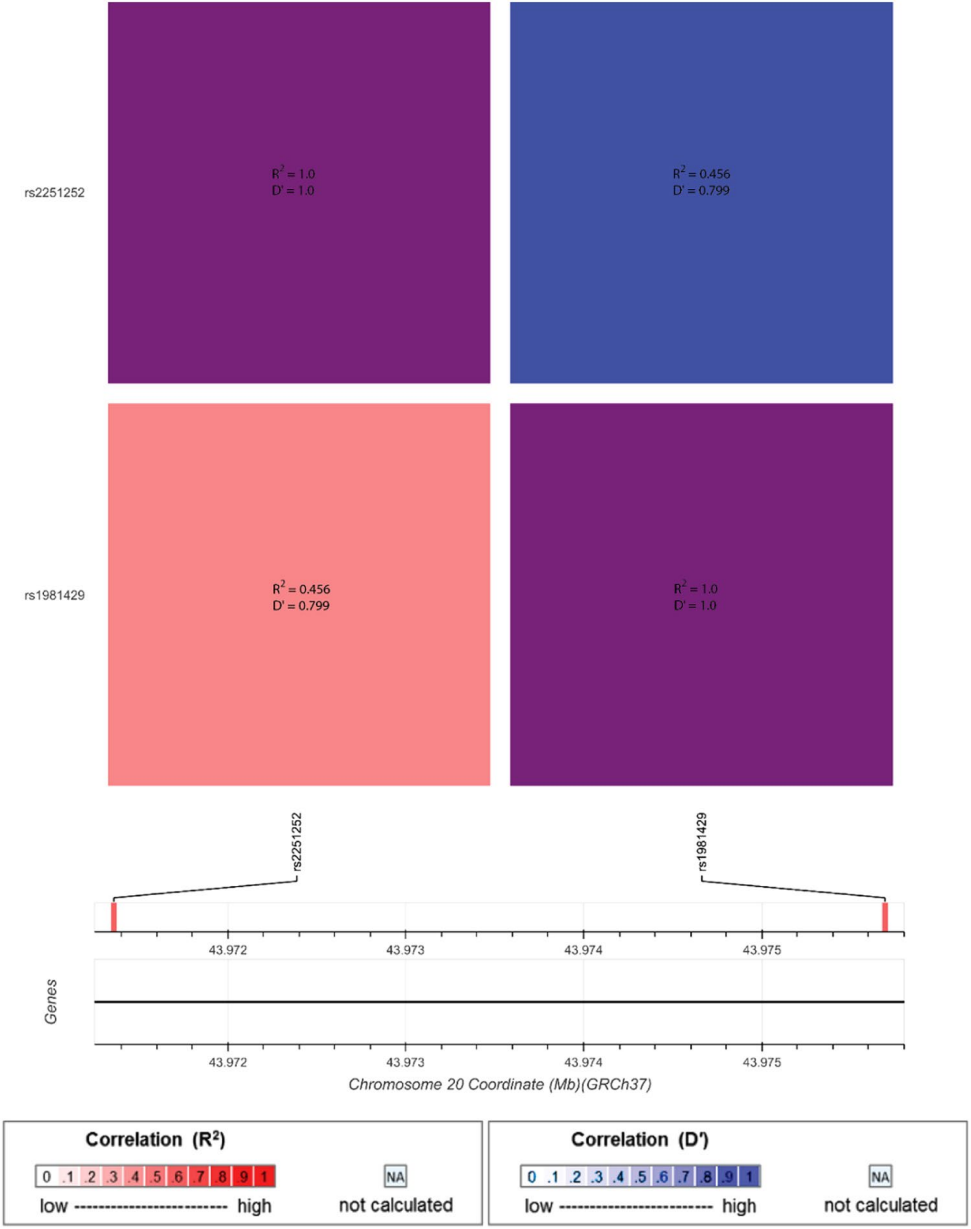
Linkage disequilibrium (LD) plot of SDC4-rs1981429 and SDC4-rs2251252 analysis generated using LD Matrix Tool on LD Link, National Cancer Institute (Karczewski et al. 2020; Machiela and Chanock 2015). Bright-red (R2) and bright-blue (D’) colours indicate higher correlation between single-nucleotide polymorphisms (SNPs), while white indicates no correlation. The purple colour in the top left and bottom right, and the coral pink in the bottom left and the cornflower blue in the top right corners indicate minimal, inconsequential correlation for the purposes of this study. The correlation values of R2 = 0.456 and D’ = 0.799 indicate no LD between these SNPs in an Australian Caucasian population

**Fig. 3:**
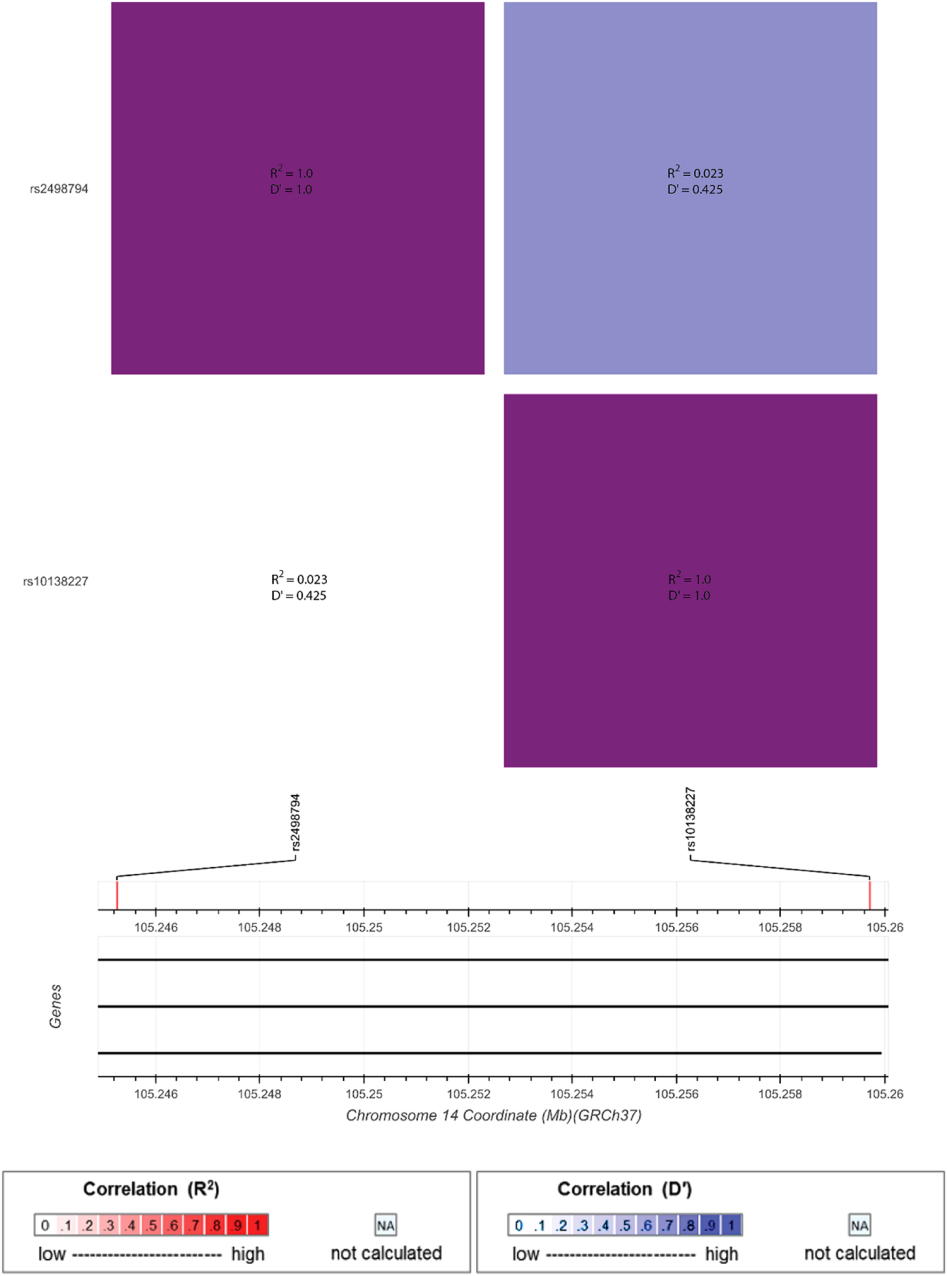
Linkage disequilibrium (LD) plot of AKT1-rs2498794 and AKT1-rs10138227 analysis generated using LD Matrix Tool on LD Link, National Cancer Institute (Karczewski et al. 2020; Machiela & Chanock 2015). Bright-red (R^2^) and bright-blue (D’) colours indicate higher correlation between single-nucleotide polymorphisms (SNPs), while white indicates no correlation. The magenta colour in the top left and bottom right, and the pale pink in the bottom left and the light purple in the top right corners indicate minimal, inconsequential correlation for the purposes of this study. The correlation values of R^2^ = 0.023 and D’ = 0.425 indicate no LD between these SNPs in an Australian Caucasian population

*SDC4* is located on chromosome 20 (Fig. 2) and AKT1 is located on chromosome 14 (Fig. 3); linkage disequilibrium (LD) analysis was performed using LD Matrix Tool on LD Link, National Cancer Institute website (Machiela and Chanock 2015). The analysis showed no LD present in an Australian Caucasian population between *SDC4*-rs1981429 and *SDC4*-rs2251252 or *AKT1*-rs2498794 and *AKT1*-rs10138227. The *ATM* gene is located on chromosome 11. LD information was not available for *ATM*-rs228590 and *ATM*-rs35098825 due to the rarity of *ATM*-rs35098825; therefore, an LD plot could not be generated.

## Results

### *SDC4*-rs1981429

Chi-square analysis of *SDC4*-rs1981429 revealed a significant association at both the genotypic and allelic level (genotype: *p* = 0.015; allele: *p* = 0.017) in the Genomics Research Centre breast cancer (GRC-BC) case–control population, all of which were in HWE (control: *p* = 0.057; case: *p* = 0.807). The OR (1.353) suggests that the presence of the minor allele C increases the risk of BC.

A replication study was performed for *SDC4*-rs1981429 in the independent Griffith University–Cancer Council Queensland Breast Cancer Biobank (GU-CCQ BB) population and analysed against the more accurately age and sex-matched 1000 Genomes Project and the more extensive gnomAD databases. Results revealed significant associations between *SDC4*-rs1981429 and BC on both the genotypic and allelic level against the genomic databases the 1000 Genomes Project data (genotype: *p* = 0.009; allele: *p* = 0.011) and gnomAD (genotype: *p* = 0.004; allele: *p* = 0.001). The control populations followed HWE (1000 Genomes Project control: *p* = 0.549; gnomAD control: *p* = 0.933), while the case population *p*-value suggests a deviation from HWE (*p* = 0.005). The OR suggests that the minor allele C increases the risk of BC development (1000 Genomes Project OR = 1.287; gnomAD OR = 1.270). Results are summarised in Table 4.

**Table 4:** Results for SDC4-rs1981429 in the Genomics Research Centre breast cancer (GRC-BC) case–control population and in the Griffith University–Cancer Council Queensland Breast Cancer Biobank (GU-CCQ BB) replication population. The cohorts were analysed using controls extracted from the 1000 Genomes Project and gnomAD databases. Global allelic HapMap frequencies are shown. Analysis revealed a significant association between SDC4-rs1981429 and BC in all statistical tests, with the presence of the minor allele C increasing the risk of BC development. The odds ratio (OR) for the GU-CCQ BB population are displayed next to the relevant control databases against which cases were analysed. The OR values indicate an increased risk associated with the C allele that is present in the case population

*SDC4*-rs1981429									
	Genotypes					Alleles			
	AA (%)	AC (%)	CC (%)	*p*-value	HWE	A (%)	C (%)	*p*-value	OR
GRC-BC cases (*n* = 270)	57 (26.54)	137 (51.15)	76 (22.31)	**0.015**	0.807	251 (46.48)	289 (53.52)	**0.017**	1.353
GRC-BC controls (*n* = 190)	63 (33.16)	81 (42.63)	46 (24.21)		0.057	207 (54.47)	173 (45.52)		
GU-CCQ BB cases (*n* = 355)	90 (25.35)	150 (42.25)	115 (32.39)		0.005	330 (46.48)	380 (53.52)		
1000 Genomes Project Controls (*n* = 404)	118 (29.21)	195 (48.27)	91 (22.52)	**0.009**	0.549	431 (53.34)	377 (46.66)	**0.011**	1.287
gnomAD controls (*n* = 9520)	2656 (27.9)	4741 (49.8)	2123 (22.3)	**0.004**	0.933	10,053 (52.8)	8987 (47.2)	**0.001**	1.270
Global HapMap frequency (%)						57.82	42.18		

Significant *p*-values of < 0.05 are highlighted in bold

### *SDC4*-rs2251252

No significant association was found between *SDC4*-rs2251252 and BC on a genotypic or allelic level (genotype: *p* = 0.075; allele: *p* = 0.813) in the GRC-BC case–control population. The control population followed HWE, while the case population deviated from HWE (control: *p* = 0.742; case: *p* = 0.005).

A replication study was performed for *SDC4*-rs2251252 in GU-CCQ BB population against the 1000 Genomes Project and gnomAD databases. Results revealed no significant association between *SDC4*-rs2251252 and BC on a genotypic or allelic level when analysed using both the 1000 Genomes Project data (genotype: *p* = 0.984; allele: *p* = 0.897) and gnomAD (genotype: *p* = 0.876; allele: *p* = 0.799). Both control and case populations followed HWE (1000 Genomes Project control: *p* = 0.763; gnomAD control: *p* = 0.933; case: *p* = 0.662). Results are summarised in Table 5.

**Table 5:** Results for SDC4-rs2251252 in the Genomics Research Centre breast cancer (GRC-BC) case–control population and in the Griffith University–Cancer Council Queensland Breast Cancer Biobank (GU-CCQ BB) replication population, analysed using controls extracted from the 1000 Genomes Project and gnomAD database. Global allelic HapMap frequencies are shown. The odds ratio (OR) values for the GU-CCQ BB population are displayed next to the relevant control database against which cases were analysed. Analysis revealed no significant association between SDC4-rs2251252 and BC

*SDC4*-rs2251252									
	Genotypes				HWE	Alleles			
	GG (%)	AG (%)	AA (%)	*p*-value		G (%)	A (%)	*p*-value	OR
GRC-BC cases (*n* = 178)	69 (38.76)	68 (38.2)	41 (23.03)	0.075	0.005	206 (57.9)	150 (42.1)	0.813	0.993
GRC-BC controls (*n* = 152)	48 (31.58)	77 (50.68)	27 (17.76)		0.742	173 (56.9)	131 (43.1)		
GU-CCQ BB cases (*n* = 342)	107 (31.29)	165 (48.25)	70 (20.47)		0.662	379 (55.41)	305 (44.59)		
1000 Genomes Project Controls (*n* = 404)	124 (30.7)	197 (48.76)	83 (20.54)	0.984	0.763	445 (55.07)	363 (44.93)	0.897	0.988
gnomAD controls (*n* = 7481)	2336 (31.23)	3692 (49.35)	1453 (19.42)	0.876	0.933	8364 (55.9)	6598 (44.1)	0.799	1.023
Global HapMap frequency (%)						54.7	45.3		

### *AKT1*-rs2498794

Genotyping analysis of *AKT1*-rs2498794 in the GRC-BC case–control cohort determined that the control population was in HWE while the case population deviated from HWE (control: *p* = 0.254; case: *p* = 0.044). Chi-square analysis showed no significant association between *AKT1*-rs2498794 and BC at either the genotypic (*p* = 0.052) or allelic (*p* = 0.458) level.

A replication study was performed for *AKT1*-rs2498794 in the GU-CCQ BB cohort and were analysed against 1000 Genomes Project and gnomAD databases. Results revealed no significant association between *AKT1*-rs2498794 and BC using the 1000 Genomes Project data (genotype: *p* = 0.057; allele: *p* = 0.728). A significant association was found on a genotypic level when the results were analysed using the gnomAD database (p = 0.005) but no significance was found on an allelic level (allele: *p* = 0.356). Both control populations followed HWE, while the case population deviated from HWE (1000 Genomes Project control: *p* = 1; gnomAD control: *p* = 0.963; case: *p* = 0.002). Results are summarised in Table 6.

**Table 6:** Results for AKT1-rs2498794 in the Genomics Research Centre breast cancer (GRC-BC) case–control population and in the Griffith University–Cancer Council Queensland Breast Cancer Biobank (GU-CCQ BB) replication population, analysed using controls extracted from the 1000 Genomes Project and gnomAD database. Global allelic HapMap frequencies are shown. Analysis revealed no significant association between AKT1-rs2498794 and breast cancer (BC) in the GRC-BC population or the GU-CCQ BB population when analysed against 1000 Genomes Project controls. The odds ratio (OR) values for the GU-CCQ BB population are displayed next to the relevant control database against which cases were analysed. Analysis of the GU-CCQ BB population against the gnomAD controls revealed a significant association between AKT1-rs2498794 and BC, potentially due to the large number of control data

*AKT1*-rs2498794									
	Genotypes				HWE	Alleles			
	AA (%)	AG (%)	GG (%)	*p*-value		A (%)	G (%)	*p*-value	OR
GRC-BC cases (*n* = 255)	62 (24.32)	144 (56.47)	49 (19.22)	0.052	0.044	268 (52.55)	242 (47.45)	0.458	1.088
GRC-BC controls (*n* = 201)	65 (32.34)	91 (45.27)	45 (22.39)		0.254	221 (54.98)	181 (45.02)		
GU-CCQ BB cases (*n* = 365)	86 (23.56)	213 (58.36)	66 (18.08)		0.002	385 (52.74)	345 (47.26)		
1000 Genomes Project Controls (*n* = 404)	116 (28.71)	201 (49.75)	87 (21.53)	0.057	1.000	433 (53.59)	375 (46.41)	0.728	1.026
gnomAD controls (*n* = 7667)	1993 (26)	3834 (50)	1840 (24)	**0.005**	0.963	7820 (51.00)	7514 (49.00)	0.356	0.922
Global HapMap frequency (%)						43.07	56.93		

Significant *p*-values of < 0.05 are highlighted in bold

### *AKT1*-rs10138227

No significant association was found between *AKT1*-rs10138227 and BC at a genotypic (*p* = 0.197) or allelic level (*p* = 0.494) in the GRC-BC case–control population. The control population followed HWE, while the case population deviated from HWE (control: *p* = 0.171; case: *p* = 0.013).

A replication study was performed for *AKT1*-rs10138227 in the GU-CCQ BB population and were analysed using 1000 Genomes Project and the gnomAD database. Results revealed no significant association between *AKT1*-rs10138227 and BC at a genotypic or allelic level when analysed using both the 1000 Genomes Project data (genotype: *p* = 0.062; allele: *p* = 0.678) and gnomAD (genotype: *p* = 0.132; allele: *p* = 0.913). Both control populations followed HWE, while the case population had a slight deviation from HWE (1000 Genomes Project control: *p* = 0.375; gnomAD control: *p* = 0.888 case: *p* = 0.041). Results are summarised in Table 7.

**Table 7:** Results for AKT1-rs10138227 in the Genomics Research Centre breast cancer (GRC-BC) case–control population and in the Griffith University–Cancer Council Queensland Breast Cancer Biobank (GU-CCQ BB) replication population, analysed using controls extracted from the 1000 Genomes Project and gnomAD. Global allelic HapMap frequencies are shown. The odds ratio (OR) values for the GU-CCQ BB population are displayed next to the relevant control database against which cases were analysed. Analysis revealed no significant association between AKT1-rs10138227 and breast cancer

*AKT1*-rs10138227									
	Genotypes				HWE	Alleles			
	CC (%)	CT (%)	TT (%)	*p*-value		C (%)	T (%)	*p*-value	OR
GRC-BC cases (*n* = 253)	181 (71.54)	52 (20.55)	20 (7.91)	0.197	0.013	414 (81.81)	92 (18.18)	0.494	1.224
GRC-BC controls (*n* = 176)	126 (71.59)	43 (24.43)	7 (3.98)		0.171	295 (83.81)	57 (16.19)		
GU-CCQ BB cases (*n* = 350)	256 (73.14)	92 (26.29)	2 (0.57)		0.041	604 (86.29)	96 (13.71)		
1000 Genomes Project Controls (*n* = 404)	308 (76.24)	87 (21.53)	9 (2.23)	0.062	0.375	703 (87)	105 (13)	0.678	1.017
gnomAD controls (*n* = 7886)	5850 (74.18)	1886 (23.92)	150 (1.9)	0.132	0.888	13,586 (86.14)	2186 (13.86)	0.913	0.951
Global HapMap frequency (%)						83.55	16.45		

### *ATM*-rs228590

Genotyping analysis of the *ATM*-rs228590 in the GRC-BC case–control cohort determined that the control population was in HWE while the case population deviated slightly from HWE (control: *p* = 0.346; case: *p* = 0.015). Chi-square analysis determined a significant association between *ATM*-rs228590 and BC. The observed positive association was at both the allelic (*p* = 3 × 10^–5^) and genotypic levels (*p* = 1.3 × 10^–5^). An OR of 1.813 suggested an increased risk of disease associated with the minor T allele.

A replication study was performed for *ATM*-rs228590 in the GU-CCQ BB population and were analysed against the 1000 Genomes Project and gnomAD databases as controls. Results revealed a significant association between *ATM*-rs228590 and BC on both genotypic and allelic level when analysed using both the 1000 Genomes Project data (genotype: *p* = 3.36 × 10^–10^; allele: *p* = 8.1 × 10^–10^) and gnomAD (genotype: *p* = 1.4 × 10^–10^; allele: *p* = 7.3 × 10^–8^). The control populations followed HWE, while the case population showed a deviation from HWE (1000 Genomes Project control: *p* = 0.749; gnomAD control: *p* = 0.915; case: *p* = 0.003). The calculated OR suggests that the minor allele C in *ATM*-rs228590 increases the risk of BC development (1000 Genomes Project OR = 1.900; gnomAD OR = 1.474). Results are summarised in Table 8.

**Table 8:** Results for ATM-rs228590 in the Genomics Research Centre breast cancer (GRC-BC) case–control population and in the Griffith University–Cancer Council Queensland Breast Cancer Biobank (GU-CCQ BB) replication population, analysed using controls extracted from the 1000 Genomes Project and gnomAD. Global allelic HapMap frequencies are shown. The odds ratio (OR) values for the GU-CCQ BB population are displayed next to the relevant control database against which cases were analysed. Analysis revealed a significant association between ATM-rs228590 and breast cancer (BC) in all analyses, with the presence of the minor allele T increasing the risk of BC development

*ATM*-rs228590									
	Genotypes				HWE	Alleles			
	CC (%)	CT (%)	TT (%)	*p*-value		C (%)	T (%)	*p*-value	OR
GRC-BC cases (*n* = 255)	58 (22.75)	106 (41.57)	91 (35.69)	**1.3 × 10**^**–5**^	0.015	222 (43.53)	288 (56.47)	**3 × 10**^**–5**^	1.813
GRC-BC controls (*n* = 173)	56 (32.37)	91 (52.6)	26 (15.03)		0.346	203 (58.67)	143 (41.33)		
GU-CCQ BB cases (*n* = 361)	92 (25.48)	151 (41.83)	118 (32.69)		0.003	335 (46.4)	387 (53.6)		
1000 Genomes Project Controls (n = 404)	157 (38.86)	193 (47.77)	54 (13.37)	**3.36 × 10**^**–10**^	0.749	507 (62.75)	301 (37.25)	**8.1 × 10**^**–10**^	1.900
gnomAD controls (n = 12,957)	4133 (31.9)	6375 (49.2)	2449 (18.9)	**1.4 × 10**^**–10**^	0.915	14,641 (56.5)	11,273 (43.5)	**7.3 × 10**^**–8**^	1.474
Global HapMap frequency (%)						46.04	53.96		

Significant *p*-values of < 0.05 are highlighted in bold

### *ATM*-rs35098825

No significant association was found between *ATM*-rs35098825 and BC in the GRC-BC case–control population. All samples were found to be homozygous for the G allele, and, therefore, statistical comparisons could not be made between the genotypes.

A replication study was then performed in the GU-CCQ BB population against the gnomAD database only as the controls. Control data were not available in the 1000 Genomes Project due to the rarity of *ATM*-rs35098825. Results obtained from the GU-CCQ BB population revealed no significant association between *ATM*-rs35098825 and BC. Results are summarised in Table 9.

**Table 9:** Results for ATM-rs35098825 in the Genomics Research Centre breast cancer (GRC-BC) case–control population and in the Griffith University–Cancer Council Queensland Breast Cancer Biobank (GU-CCQ BB) replication population, analysed using controls extracted from gnomAD. Control data from the 1000 Genomes Project and HapMap frequencies were not available for this rare SNP. All samples were found to be homozygous for the G allele and no significant association between ATM-rs35098825 and breast cancer was observed

*ATM*-rs35098825									
	Genotype				HWE	Allele			
	GG (%)	AG (%)	AA (%)	*p*-value		G (%)	A (%)	*p*-value	OR
GRC-BC cases (*n* = 232)	232 (100)	0 (0)	0 (0)	N/A	N/A	464 (100)	0 (0)	N/A	N/A
GRC-BC controls (*n* = 153)	153 (100)	0 (0)	0 (0)		N/A	306 (100)	0 (0)		
GU-CCQ BB cases (*n* = 361)	361 (100)	0 (0)	0 (0)	N/A	N/A	722 (100)	0 (0)	N/A	N/A
gnomAD controls (*n* = 113,720)	113,720 (100)	0 (0)	0 (0)		N/A	227,440 (100)	0 (0)		

## Discussion

In this study, we examined two SNPs in the gene encoding the HSPG core protein, *SDC4*, and four SNPs in the HSPG-related genes *AKT1* and *ATM* for associations with BC risk. Our data indicated that the SNPs *SDC4*-rs1981429 and *ATM-*rs228590 are associated with BC risk in an Australian Caucasian population. The minor alleles, C in *SDC4-*rs1981429 and T in *ATM-*rs228590, were found to be significantly more common in BC patients, increasing the risk of BC development. *SDC4*-rs2251252, *AKT1*-rs2498794, *AKT1*-rs10138227 and *ATM*-rs35098825 were examined in the same population cohorts and were not found to be associated with BC risk or susceptibility (Bonin et al. 2019).

The initial study was conducted in the Genomics Research Centre (GRC) breast cancer (GRC-BC) population and was replicated in an independent Griffith University–Cancer Council Queensland Breast Cancer Biobank (GU-CCQ BB) population to increase the case numbers. As this was a case-only population, genotypes were extracted from populations in the 1000 Genomes Project to provide extended control data for *SDC4*-rs1981429, *SDC4*-rs2251252, *AKT1*-rs2498794, *AKT1*-rs10138227 and *ATM*-rs228590 (Genomes Project Consortium et al. 2015). For *ATM*-rs35098825, control data were extracted from gnomAD (Karczewski et al. 2020) as this information was not available in the 1000 Genomes Project due to the rarity of the SNP.

We hypothesised that the presence of the minor allele C in *SDC4*-rs1981429 would increase the risk of BC development. The more common allele A has been linked to increased longevity in a previous study, while the minor allele C was shown to decrease longevity (Rose et al. 2015). Analysis of *SDC4*-rs1981429 results in both the GRC-BC cohort and GU-CCQ BB populations revealed that the common A allele conferred a protective influence against developing BC, while the minor allele C significantly increased risk of developing BC. Previously, *SDC4*-rs1981429 has been shown to have a regulatory function in signalling pathways and gene expression (Rose et al. 2015). The presence of the minor allele C in *SDC4*-rs1981429 may correct the inhibitory interaction of *SDC4* in pathways such as PI3K/AKT, resulting in more normalised gene expression and cellular proliferation, subsequently reducing the risk of developing BC. Functional studies would be beneficial to fully elucidate the role of this SNP and *SDC4* in the PI3K/AKT signalling pathway and BC.

Previous findings have linked SDC expression to BC-related signalling pathways, such as Wnt-pathways (Okolicsanyi et al. 2015; Tkachenko et al. 2005). A study in 2015 by Okolicsanyi et al. found that *SDC1* promotes tumourigenesis and angiogenesis in ER-negative breast tumours, with *SDC1* SNPs correlating with an increased risk of BC in an Australian Caucasian population (Okolicsanyi et al. 2015). Recent unpublished data by Pham et al. (2022) has also shown that the *SDC4* core protein has a significant regulatory effect on cell proliferation in BC through enhancement of the HS side chain.

**Fig. 4:**
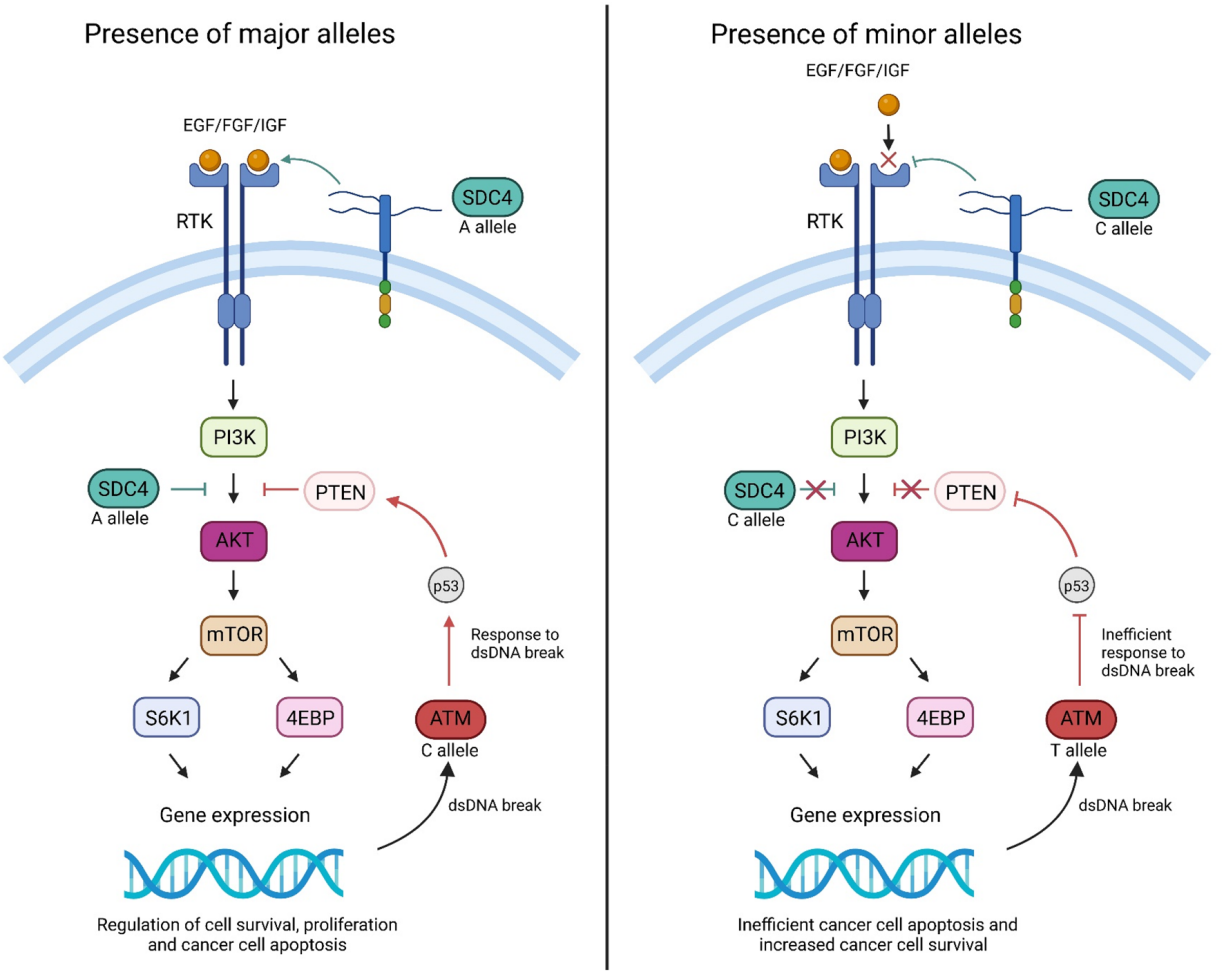
Hypothetical model of the Phosphoinositide 3-kinase/AKT Serine/Threonine-Protein Kinase (PI3K/AKT) signalling pathway in the presence of the major and minor alleles of the syndecan-4 (SDC4) and ATM serine/threonine kinase (ATM) single-nucleotide polymorphisms, SDC4-rs1981429 and ATM-rs228590. The minor allele C in SDC4-rs1981429 may partially inhibit the binding of growth factors, including Epidermal Growth Factor (EGF), Fibroblast Growth Factor (FGF) and Insulin-like Growth Factor (IGF), that activate tyrosine kinase receptors (RKT). This may result in downregulation of the downstream signalling cascade, decreasing the functions of the PI3K/AKT pathway. This may result in aberrant responses to abnormal cellular proliferation and cancer cell apoptosis. SDC4 also regulates the pathway downstream of PI3K by inhibiting AKT. This inhibitory effect may be lost in the presence of the minor allele C in SDC4-rs1981429, resulting in aberrant behaviour of the downstream cascade. Meanwhile, the presence of the minor allele T in ATM-rs228590 may result in an inefficient response when double-stranded DNA (dsDNA) breaks are detected and, therefore, incomplete activation of the ATM gene. This inhibits stimulation of the tumour protein p53 (p53), which in turn inhibits Phosphatase and Tensin homolog (PTEN, which is known to inhibit the function of the PI3K/AKT pathway. If PTEN fails to regulate the downstream functions of AKT and mammalian target of rapamycin (mTOR), the downstream proteins, including Ribosomal protein S6 kinase beta-1 (S6K1) and 4E-binding protein (4EBP) will be abnormally expressed. This may result in anomalous gene expression, resulting in abnormal tumour cell proliferation, inefficient cancer cell apoptosis and increased breast cancer risk

Interestingly, *SDC4*-rs1981429 is an intronic, non-coding variant. Previously, *SDC4* SNPs have been found to influence the binding of growth factors, including FGF and EGF (Corti et al. 2013; Karczewski et al. 2020; Machiela & Chanock 2015; Schadt et al. 2008). Our findings indicate that while not directly functional, *SDC4*-rs1981429 has a significant effect on cancer-related pathways, increasing BC risk. The presence of the minor allele may impede the binding of growth factors such as FGF, inhibiting the upstream action of RTK as demonstrated in the schematic presented in Fig. 4. If RTK stimulates the downstream steps of the PI3K/AKT pathway via complex regulation of PI3K (Corti et al. 2013; Gross and Rotwein 2016; Karczewski et al. 2020; Machiela and Chanock 2015; Schadt et al. 2008); by inhibiting RTK, *SDC4*-rs1981429 may downregulate the PI3K/AKT pathway, resulting in increased cancer cell survival via inefficient activation of cellular responses to DNA damage and inhibiting apoptosis of precancerous or cancerous cells. *SDC4* also regulates the pathway downstream of PI3K and upstream of AKT. This regulatory effect may be lost in the presence of *SDC4*-rs1981429, resulting in aberrant behaviour of the downstream cascade.

No statistical significance was found between *AKT1*-rs10138227 and BC in the GRC-BC case–control cohort or the GU-CCQ BB population when analysed against controls extracted from the 1000 Genomes Project; however, the p-values from these analyses (GRC-BC case control: *p* = 0.052; GU-CCQ BB and 1000 Genomes Project: *p* = 0.057) trend towards statistical significance. A significant association was found when the GU-CCQ BB population results were analysed against the larger gnomAD database population control data. The significance found may be due to the larger control population being available through gnomAD; additionally, the control data extracted from the 1000 Genomes Project was matched more closely to the demographic data in our GU-CCQ BB case-only population in terms of gender and European origin. *AKT1*-rs10138227 has previously been associated with cancer status and smoking duration and may be associated with psychiatric disorders (Nishizawa et al. 2015). Likewise, *AKT1*-rs2498794 may increase the risk of endometrial cancer (Painter et al. 2016). A larger population study should be undertaken to further explore the implications of the *AKT1* variants in BC.

Like *SDC4*-1981429, *ATM-*rs228590 is an intronic and non-coding variant. In the PI3K/AKT pathway, the *ATM* gene detects dsDNA damage and initiates repair of that damage (Zhang et al. 2017). Analysis of *ATM-*rs228590 results in both the GRC-BC cohort and GU-CCQ BB population showed that the presence of the minor allele T appears to increase the risk of BC. *ATM-*rs228590 is in intron 1 of *ATM*, located in the promoter region of the gene and is known to influence the binding of transcription factors (Xiong et al. 2013). Xiong et al. (2013) found that the presence of the T allele in *ATM-*rs228590 increased the risk of severe radiation pneumonitis in patients with non-small cell lung cancer (Kuba et al. 2015; Xiong et al. 2013). Our study suggests that the T allele in *ATM-*rs228590 disrupts the inhibitory effect of *ATM*, potentially by altering transcription. This in turn inhibits *PTEN*, which is known to inhibit the function of the PI3K/AKT pathway (Bonin et al. 2019). Aberrant activation of the pathway has been shown to frequently contribute to cancer development, including breast, colon and rectal and haematological cancers (Yang et al. 2019). *ATM*-rs228590 may, therefore, upregulate the function of the pathway, thus resulting in increased cancer cell proliferation, increasing the development of BC. *ATM-*rs228590 is a promoter SNP, which means that its presence may change the encoding of the ATM protein, thus altering the ability of the protein to react to a dsDNA break. This in turn would inhibit cellular responses to DNA damage or trigger an incomplete response, decreasing the potency of PI3K/AKT pathway of response to malignant cellular changes.

These intronic SNPs in the *SDC4* and *ATM* genes may, therefore, disrupt or suppress the binding or function of pathways such as the PI3K/AKT pathway, altering gene expression and increasing the risk of BC development. Due to their significant role in numerous cellular mechanisms closely associated with cancers, HSPGs, such as *SDC4*, and HSPG-related genes such as *AKT1* and *ATM* present an exciting target for BC diagnostics and macromolecular drug therapies.

## Conclusion

The involvement of SDCs and signalling pathways in BC development is a fascinating area of research. Here, we investigated two *SDC4*, two *AKT1* and two *ATM* SNPs and their effect on BC risk. This study suggests that the tested *SDC4-*rs1981429 marker may have a protective role in BC progression and development, while the *ATM-*rs228590 marker may increase BC risk. We expected to see an increased risk of BC development associated with the *SDC4*, *AKT1* and *ATM* markers. Our current study supports this hypothesis with a positive association observed between the C allele of *SDC4*-rs1981429 and BC. In addition, the presence of the T allele in *ATM-*rs228590 demonstrated an increased risk of BC development. Meanwhile, *SDC4*-rs2251252, *AKT1-*rs2498794, *AKT1*-rs10138227 and *ATM-*rs35098825 showed no association with disease in the studied population cohorts. *SDC4*-rs1981429 and *ATM-*rs228590 SNPs may be potential biomarkers for early BC detection, in turn improving patient outcomes. Building a robust molecular profile by identifying key BC biomarkers in HSPGs and HSPG-related genes will improve risk evaluation and early diagnosis as well as devising personalised anticancer approaches. Understanding the signalling pathways and the genetic changes leading to dysregulation will provide insight into BC development and progression, providing an opportunity to target abnormalities in these genes and regulate BC advancement.

## Data Availability

Not applicable.
